# Prescription, Compliance, and Burden Associated with Salt-Restricted Diets in Heart Failure Patients: Results from the French National OFICSel Observatory

**DOI:** 10.3390/nu14020308

**Published:** 2022-01-12

**Authors:** Thibaud Damy, Véronique Benedyga, Théo Pezel, Emmanuelle Berthelot, Jacques Gauthier, Gilbert Habib, Marie-Christine Iliou, Jean-François Aupetit, Guillaume Baudry, Pascal De Groote, Damien Logeart, Laure Chaufourier, Vlad Ciobotaru, Françoise Pousset, Florence Beauvais, Fabrice Bauer, Florian Zores, Olivier Lairez, Kevin Richard, Luc Hittinger, Emmanuel Teiger, Charles Taieb, Etienne Audureau

**Affiliations:** 1 Centre de Référence Amyloses Cardiaques et des Cardiomyopathies, Cardiology Department, CHU Henri Mondor, APHP, 94000 Créteil, France; benedyga@orange.fr (V.B.); luc.hittinger@aphp.fr (L.H.); emmanuel.teiger@aphp.fr (E.T.); 2 AP-HP Public Health Department, CHU Henri Mondor, 94000 Créteil, France; etienne.audureau@aphp.fr; 3 Medicine Teaching School, University Paris Est Créteil, INSERM, IMRB, 94010 Créteil, France; 4 Cardiology Department, CHU Lariboisière, APHP, 75010 Paris, France; theo.pezelccf@gmail.com (T.P.); damien.logeart@aphp.fr (D.L.); florence.beauvais@aphp.fr (F.B.); 5 Cardiology Department, CHU Bicêtre, APHP, 94250 Le Kremlin-Bicêtre, France; emmanuelle.berthelot@aphp.fr; 6 Independent Researcher, 13104 Arles, France; jchm.gauthier@orange.fr; 7 Cardiology Department, Hôpital de la Timone, APHM and Aix Marseille University, APHM, 13000 Marseille, France; gilbert.habib3@gmail.com; 8 Cardiac Rehabilitation Department, Hôpital Corentin Celton, APHP, 92130 Issy Les Moulineaux, France; marie-christine.iliou@aphp.fr; 9 Cardiology Department, CH Saint Joseph-Saint Luc, 69007 Lyon, France; jfaupetit@ch-stjoseph-stluc-lyon.fr; 10 Heart Failure Department, Louis Pradel Hospital, Hospices Civils de Lyon, 69500 Bron, France; baudryguillaume@gmail.com; 11 Service de Cardiologie, Institut Pasteur de Lille, Inserm U1167, CHU Lille, 59000 Lille, France; pascal.degroote@chru-lille.fr; 12 Independent Researcher, 14600 Honfleur, France; laurechaufourier@gmail.com; 13 Department for Valvular and Structural Cardiopathy Exploration, Hôpital Privé Les Franciscaines-Elan, 30000 Nimes, France; vciobotaru@yahoo.fr; 14 Cardiology Departement, Hôpital de la Pitié Salpêtrière, APHP, 75013 Paris, France; f.pousset@aphp.fr; 15 Cardiology Department, CHU Gabriel-Montpied, 63000 Clermont-Ferrand, France; fabrice.bauer@chu-rouen.fr; 16 Independent Researcher, 67000 Strasbourg, France; florian.zores@gmail.com; 17 Cardiology Department, CHU Rangueil, 31059 Toulouse, France; lairez@gmail.com; 18 Cardiac Rehabilitation Department, CHU Albert Chenevier, 94000 Créteil, France; kevin.richard@aphp.fr; 19 Emma Clinic, 94300 Vincennes, France; charles.taieb@emma.clinic

**Keywords:** heart failure, patient education, restricted diets, salt diet, OFICSel observatory, patient burden

## Abstract

(1) Background: There is much debate about the use of salt-restricted diet for managing heart failure (HF). Dietary guidelines are inconsistent and lack evidence. (2) Method: The OFICSel observatory collected data about adults hospitalised for HF. The data, collected using study-specific surveys, were used to describe HF management, including diets, from the cardiologists’ and patients’ perspectives. Cardiologists provided the patients’ clinical, biological, echocardiography, and treatment data, while the patients provided dietary, medical history, sociodemographic, morphometric, quality of life, and burden data (burden scale in restricted diets (BIRD) questionnaire). The differences between the diet recommended by the cardiologist, understood by the patient, and the estimated salt intake (by the patient) and diet burden were assessed. (3) Results: Between March and June 2017, 300 cardiologists enrolled 2822 patients. Most patients (90%) were recommended diets with <6 g of salt/day. Mean daily salt consumption was 4.7 g (standard deviation (SD): 2.4). Only 33% of patients complied with their recommended diet, 34% over-complied, and 19% under-complied (14% unknown). Dietary restrictions in HF patients were associated with increased burden (mean BIRD score of 8.1/48 [SD: 8.8]). (4) Conclusion: Healthcare professionals do not always follow dietary recommendations, and their patients do not always understand and comply with diets recommended. Restrictive diets in HF patients are associated with increased burden. An evidence-based approach to developing and recommending HF-specific diets is required.

## 1. Introduction

Worldwide, about 26 million people have heart failure (HF) [1,2]. HF is a major public health concern, with high and increasing rates of hospitalisation and mortality, and is associated with substantial economic burden [3,4,5]. In 2010, 159,143 French patients with HF were hospitalised [1]. The most challenging issue for HF management is reducing hospitalisations and readmissions of chronic HF patients. HF is associated with sodium and water retention, eventually resulting in excessive fluid retention in the body.

Due to the sodium retention observed, reducing the intake of sodium, through a low-salt diet, plays an essential part in HF management [6]. The main source of dietary sodium is table salt (sodium chloride). There is about 1 g of sodium in 2.5 g of salt. Daily, we require between 1 and 2 g of salt (0.4–0.8 g of sodium). Thus, a low-salt diet with a daily limit of 6 g of salt limits daily sodium consumption to <2.4 g.

However, the benefit of restricting dietary sodium in patients with HF remains controversial [6,7,8,9]. There is evidence that high-salt intake results in fluid retention, increased blood pressure, and increased cardiovascular risk [8]. However, restricting sodium intake may have a negative effect on kidney function with increased plasma levels of neurohormonal and cytokines: increasing hospital readmissions and mortality [7,10,11,12]. Indeed, a study assessed long-term effects of a moderate- (2.8 g sodium/day; 7.0 g salt/day) compared to a low-sodium diet (1.8 g sodium/day; 4.5 g salt/day). Parrinello et al. concluded that a moderate-sodium diet combined with a restricted fluid intake (1 L/day) may be more beneficial than the low-sodium diet and unlimited fluid intake usually recommended [10].

Due to the lack of evidence and consensus, HF clinical practice guidelines lack consistency and have changed over time [8,13]. In 2012, the ESC indicated that there was not enough evidence to recommend restricting dietary salt [14]. The American Heart Association (AHA), in 2013, recommended that patients with more severe HF restrict sodium to <1.5 g/day (salt to <3.75 g/day) and other HF patients to <3 g/day (salt to <7.5 g/day) [15]. The 2016 European Society of Cardiology (ESC) guidelines recommend that HF patients restrict salt consumption to 6 g/day [16]. The 2021 ESC guidelines recommend that diets with salt intake of >5 g/day should be avoided [17]. While the Canadian Cardiovascular Society (CCS) 2017 guidelines suggest a consumption of between 2 and 3 g/day [9]. More recently, in 2018, the National Heart Foundation of Australia and the Cardiac Society of Australia and New Zealand recommended that HF patients limit sodium intake to <2 g/day (salt to 5 g/day), as recommended for the general population [18]. There is, however, consensus that large, randomised studies are required to provide definitive evidence concerning the role of salt diets in treating HF patients.

When implementing lifestyle changes in patients, it is important to assess the associated burden perceived by the patient. Indeed, increased burden may result in non-compliance, both dietary and with medication, inducing various complications. However, because of the lack of appropriate measurement instruments, this issue has mostly remained unaddressed. The burden scale in restricted diets (BIRD) questionnaire was recently developed and validated to estimate the patient’s perceived burden associated with implementing a sodium-restricted diet [19], but detailed information on perceived burden in HF patients is still lacking.

The OFICSel observatory collected data to describe the characteristics of HF patients and treatments, including how restricted-salt diet is prescribed by cardiologists and how it is understood and eventually applied by HF patients, as well as their impact on the patients’ life and perceived burden.

## 2. Method

### 2.1. Study Design

OFICSel was a non-interventional, observational, cross-sectional, and multicentre observatory. About 1000 French cardiologists were solicited to participate in this observatory. Patients older than 18 years of age and hospitalised, at least once, for heart failure during the previous 5 years were eligible. Patients unable to understand French were ineligible.

The study was performed in accordance with the Declaration of Helsinki. The Comité Consultatif sur le Traitement de l’Information en matière de Recherche dans le domaine de la Santé (n°16-109, 17 February 2016) and Commission Nationale de l’Informatique et des Libertés (n°916224, 4 October 2016) approved the study. The French Society of Cardiology promoted this study.

### 2.2. Data Collection

**Figure 1:**
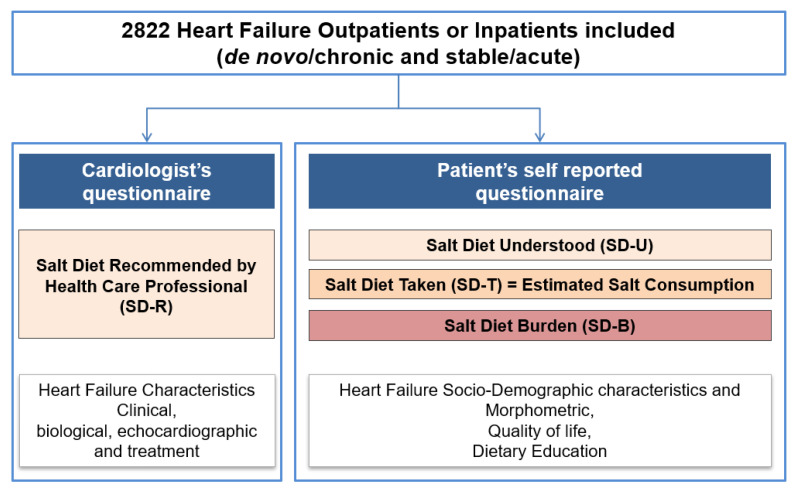
Study design.

Data were collected from patients and their cardiologists using study-specific surveys (Figure 1). The patients were blinded from their cardiologists’ responses. The patients were requested to complete a study-specific survey that collected sociodemographic, medical history and heart failure-related data, health-related quality-of-life data, and dietary data. The dietary data included the salt diet recommended (SD-R), as a specific cardiologist question, the salt diet understood (SD-U), as a specific patient question, and the salt diet taken (SD-T) and the salt diet burden (SD-B) induced by the dietary recommendations, as two independent cardiologist and patient questionnaires, as shown in Figure 1. Health-related quality of life data were collected using the Minnesota Living with Heart failure Questionnaire (MLHFQ), which comprises 21 items divided into physical, emotional, and socioeconomic effects of HF on daily life, each scored on a six-point Likert scale from 0 to 5 [20]. The MLHFQ provides an overall score (range 0 to 105 points) and two subscales/dimensions: physical (range 0 to 40) and emotional (range 0 to 25). The SD-T were estimated using a dietary instrument that we developed [21]. The instrument consists of nine questions and was specifically developed to estimate salt consumption in patients with chronic HF. The study evaluating this instrument found that daily salt consumptions estimates were of similar magnitude to those estimated by dieticians and patients: the mean difference in the estimates was 0.4 g of salt consumed per day. The patients were unaware that the study-specific survey responses were used to estimate their salt consumption. SD-B were estimated using the 12-item burden scale in restricted diets (BIRD) questionnaire, and each item scored on a 5-point Likert scale from 0 to 4 (range 0 to 48 points) [19]. Missing items were imputed using the average of the other items. BIRD questionnaires with less than seven items completed were not included in the analysis.

The cardiologist’s study-specific survey collected data concerning the patient and their HF, including the diet recommended, and clinical and disease characteristics. Data collected concerning HF included type (right, left, or global heart failure), aetiology, New York Health Association (NYHA) class, date of diagnosis, treatment (whether or not a multi-site and/or a defibrillator pacemaker was implanted), as well as electrocardiogram (ECG) and echocardiographic data (e.g., left ventricular ejection fraction (LVEF)). In addition, biological data, including *N*-terminal pro-brain-type natriuretic peptide (NT-proBNP), B-type natriuretic peptide (BNP), and/or serum creatinine levels, were collected. NT-proBNP and BNP levels were each classified into quartiles. These quartiles were then combined to obtain a unique biomarker quartile class combining NT-proBNP and BNP levels. The study-specific surveys also collected data concerning the patient’s compliance with their therapeutic education programme.

### 2.3. Study Objectives and Outcomes

This observational study comprised several main objectives, including the description of the low-salt diets recommended for French HF patients, the patients’ understanding of these diets, their adherence, perceived burden, and health-related quality of life. Moreover, we wanted to identify determinants of the patients’ compliance and understanding of the low-salt diet recommended.

### 2.4. Statistical Analysis

Continuous variables are expressed as mean with the associated standard deviation (SD) or median with interquartile range (IQR). Categorical variables are reported as number with frequency (%). Percentages were calculated relative to available data. Missing data were not imputed. Patient dietary compliances, the correlation between the SD-U, by the patient, and the estimated salt consumption (SD-T), were classified as either compliant, overcompliant, undercompliant, or unknown. The concordance between SD-R and SD-U were tested using the kappa test and displayed as Sankey plot.

Association between SD-U and BIRD questionnaire score depending on patient compliance is shown as Sankey plots. Univariate and stepwise backward multivariate linear regression modelling were used to identify independent determinants of daily estimated salt consumption (SD-T), and stepwise backward multivariate logistic regression modelling to identify determinants of the burden (SD-B) associated with SD-U (categorised according to quartiles [Q] of the BIRD questionnaire distribution: Q1–3 versus Q4).

All statistical analyses were performed using Stata version 15.1 (StataCorp, College Station, TX, USA).

## 3. Results

### 3.1. Characteristics of Heart Failure Patients

Between March and June 2017, 2822 patients were enrolled by 300 cardiologists from 180 French cardiology departments and private practices. Most patients, 1350 (53.1%), were hospitalised, with a further 283 (11.1%) in cardiac rehabilitation centres and 908 (35.7%) treated as out-patients; data were unknown for 251 and missing for 30 patients. Overall, patients were mostly male (*n* = 1978; 70.2%) and the mean age was 67.4 years (SD: 13.8). Most patients had chronic HF (*n* = 2180; 83.6). Moreover, HF was predominantly stable (*n* = 1788; 69.4%). The demographic, clinical, and biological characteristics of patients are shown in Table 1.

### 3.2. Diets Recommended (SD-R) and Understood (SD-U)

Most patients, 2618 (92.8%), were recommended a low-salt diet: predominantly with ≤6 g of salt per day (2368 patients [83.9%]). A salt diet restricted to below 3 g of salt/day was only recommended in 267 patients (9.5%). More information is provided in Table 2. In addition, 1899 patients (67.3%) understood that they were to follow a low-salt diet with ≤6 g of salt per day. This included 516 patients (18.3%) who understood that they should consume <3 g of salt/day.

### 3.3. Estimation of Salt Consumption (SD-T) and Dietary Compliance

The estimated mean daily salt consumption was 4.7 g (SD: 2.4). Dietary compliance, comparing the SD-U and the estimated salt consumption (SD-T), was as follows: 933 patients (33.1%) were compliant, 969 (34.3%) overcompliant, 525 (18.6%) undercompliant, and 395 (14.0%) with unknown compliance; see Table 2.

### 3.4. Burden (SD-B) and Quality of Life Associated with Adopting a Low-Salt Diet

SD-B associated with adopting a low-salt diet was assessed using the BIRD questionnaire. In our study, 220 BIRD questionnaires (7.8%) had <7 items completed and were not analysed. BIRD score (maximum score of 48) was on average 8.1 (SD: 8.8), with a median of 5.0 (IQR: 1–13). The mean scores for each of the 12 items of the BIRD questionnaire ranged from 0.4 to 1.1 out of 4 (5-point Likert scale); see Table 2. Patient quality of life was assessed using the HF-specific MLHFQ. In the OFICSel observatory, the average overall MLHFQ score (maximum of 105) was 35.4 (SD: 24.5). The average score for the physical dimension (maximum of 40) of the MLHFQ was 16.7 (SD: 11.7) and for the emotional dimension (maximum of 25) was 7.7 (SD: 6.6). The patients were classified into quartiles according to their burden (SD-B), using the BIRD score. Patients with a SD-B in Q1–3 (*n* = 1983) had an average BIRD score of 3.9 (SD: 3.9) compared to 21.4 (SD: 6.9) in those with a SD-B in Q4 (*n* = 619); see Table S1 in Supplementary Materials. Moreover, patients classified as SD-B in Q1–3 (*n* = 1983) had an average overall MLHFQ score of 29.6 (SD: 22.2) compared to 56.5 (SD: 19.3) for those classified in Q4 (*n* = 619). The physical dimension (/40) was on average 14.4 (SD: 11.1) in Q1–3 patients and 25.0 (SD: 9.5) in Q4 patients. Similarly, the emotional dimension (/25) was on average 6.2 (SD: 5.8) in Q1–3 patients and 13.2 (SD: 5.7) in Q4 patients.

### 3.5. Concordance between Salt Diet Recommended (SD-R) and Salt Diet Understood (SD-U)

**Figure 2:**
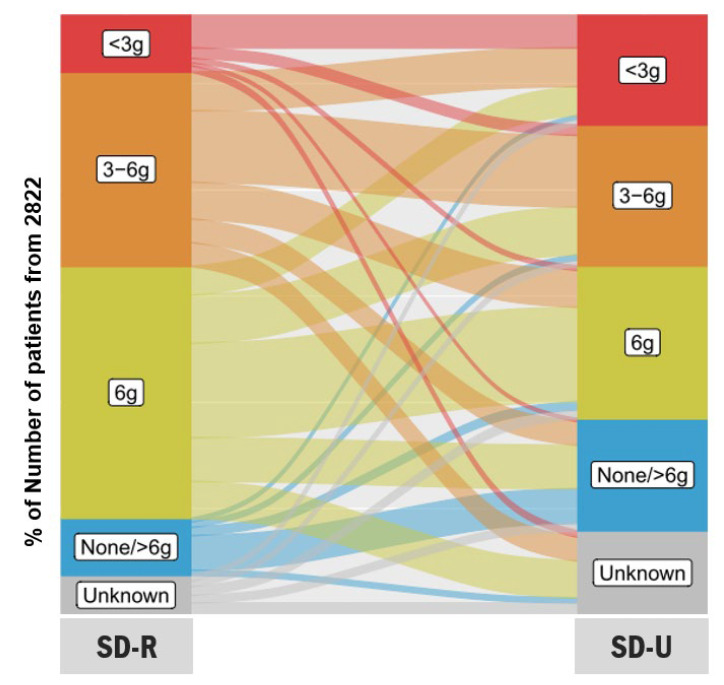
Sankey plots showing the correlation between salt diet recommended by healthcare professional (SD-R) and salt diet understood (SD-U) by the 2822 patients included in the OFICSel study.

The concordance between each SD-R and each SD-U is shown in Table 3 and displayed as Sankey plot in Figure 2. An overall agreement of 40.9% (*n* = 1153/2822) was found between SD-R and SD-U, yielding a kappa concordance coefficient of 0.234 (standard error ± 0.009). The factors associated with disagreement between the SD-R and the SD-U, i.e., overestimation and underestimation, are provided in Table S2 (Supplementary Materials). Factors associated with patients’ underestimation of salt diet were older age, living alone, high left ventricular ejection fraction, valvular HF type, and never weighing. Factors associated with patients’ overestimation of salt diet were low heart rate, high haemoglobin, lower prevalence of history of hypertension, less symptomatic HF (lower NYHA class), lower prevalence of acute HF versus stable HF and longer history of acute HF episode, higher frequency of salt diet recommended by cardiologists or dieticians, and not living alone.

### 3.6. Univariate and Multivariate Analyses Identifying Determinants of Daily Estimated Salt Consumption (SD-T)

A univariate analysis was performed, followed by a multivariate analysis, to identify determinants of estimated salt consumption (Table 4). Results of the univariate linear regression modelling of the determinants of daily estimated salt consumption (SD-T) are shown in Table S3 (Supplementary Materials). Decreased salt consumption was independently associated with the following determinants: female sex, living in a retirement home/community or alone, having chronic HF (versus de novo), having acute HF (versus stable), when the cardiologist recommended the diet, and in patients with daily, weekly, or monthly weighing (the more frequently the patients were weighed, the more salt consumption was reduced). By contrast, living in an urban environment and being a current smoker were determinants of increased salt consumption.

### 3.7. Univariate and Multivariate Analysis Identifying Determinants of Patients’ Burden Associated with Salt Diet Understood (SD-U), According to BIRD Scores

A univariate and then a multivariate analysis were performed to identify determinants of the patients’ burden (SD-B) associated with SD-U by the patients (Table 5). Complete results from the univariate logistic regression modelling analysis of determinants of the burden associated with salt diet are shown in Table S4 (Supplementary Materials). The analyses found that increased age, increased left ventricular ejection fraction, higher haemoglobin levels, increased salt consumption reported by the patient, and overcompliance with SD-U were independently associated with less burden, as perceived by the patient (assessed using BIRD scores). Patients reported more burden (higher BIRD scores) when they were female, lived in urban environments, had acute HF (rather than stable), had hypercholesterolemia, diabetes, or chronic obstructive pulmonary disease, had NYHA class III or IV, and were undercompliant with SD-U.

### 3.8. Relatedness of the Daily Estimated Salt Consumption (SD-T) with Perceived Burden Stratifying by Compliance

**Figure 3:**
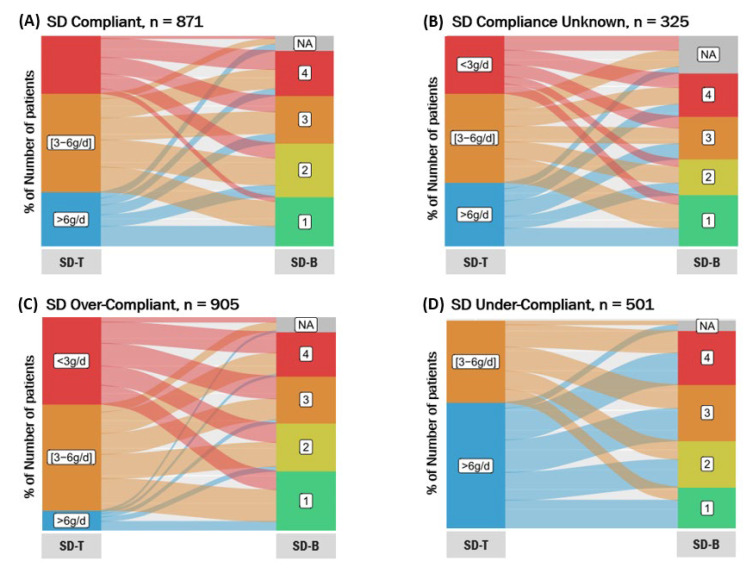
Sankey plots showing the correlation between daily salt diet taken (SD-T) and salt diet burden (SD-B) estimated using the BIRD questionnaire score depending on the compliance of the patients: (**A**) compliant; (**B**) compliance unknown; (**C**) overcompliant; (**D**) undercompliant.

Sankey plots showing the correlation between daily salt diet taken (SD-T) and salt diet burden (SD-B) estimated using the BIRD questionnaire score are shown globally in the Graphical Abstract and, depending on the compliance of the patients, in Figure 3. Briefly, patients who were compliant and overcompliant showed lower Q4 BIRD proportion than those who were undercompliant despite having a more restrictive salt diet. The larger proportion of patients with Q1 BIRD comprised those who were overcompliant.

## 4. Discussion

The OFICSel observatory is to our knowledge the first study to assess salt diets in a large and representative HF population in France. Indeed, patients were enrolled from various social and economic backgrounds and in various healthcare settings throughout France. Quantifying patient burden associated with adopting lifestyle changes, such as restricting salt intake, is challenging but important. The BIRD questionnaire was developed specifically to assess burden in HF patients adopting a salt diet [19]. The questionnaire was initially validated in 152 HF patients, with a median score of 6.5 (IQR: 2.0–14.0). In the 2822 patients enrolled in the OFICSel observatory, the mean score was 8.1 (SD: 8.8). Our study showed that the BIRD questionnaire provides valuable information from a patient’s perspective and validates this instrument in a large study.

Interestingly, 42% of patients in the OFICSel observatory were recommended salt-restricted diet (<6 g of salt/day) by their cardiologists despite the current lack of evidence. In addition, only 42% were prescribed diets with 6 g of salt per day, as recommended by the ESC in 2016 [16]. We also observed that recommending diets for HF patients, irrespective of the diet, was associated with more burden, as evidenced by an average BIRD score of only 8.1/48. However, this association does not imply that there is a causal relationship between recommending diets to HF patients and the increased burden observed. Furthermore, concerning patient quality of life, as measured using the MLHFQ, we observed an average physical dimension score of 16.7/40 and emotional dimension score of 7.7/25. These are comparable with the average 14.8 and 5.9, respectively, observed in 544 HF patients reported by Naveiro-Rilo et al. [22].

Our results highlight a clear need for patient therapeutic education concerning diet and lifestyle changes. Overall, 83.9% of our patients were recommended low-salt diets (≤6 g of salt/day). However, only 67.3% of patients understood that they should consume ≤6 g of salt/day, as recommended by the ESC guidelines (2016) [16]. If dietary changes are an important part of HF treatment, then healthcare professionals need to ensure that patients clearly understand the diet recommended. Once the patient clearly understands the diet recommended, then they need to ensure that they comply with these recommendations. Indeed, in our study, only 33% of patients complied with the diet understood.

It is vital to identify the factors associated with HF patients’ compliance with lifestyle recommendations, including those concerning salt-restricted diets. Indeed, compliance favours better health, lower mortality, and fewer hospital readmissions, and lowers healthcare costs [23,24]. Older, more educated, and health-literate patients are reportedly more compliant [24,25]. Concerning salt-restricted diets, men tend to be less compliant with diets recommended: they generally eat more and consume food with more salt [26]. Moreover, when a family member or caregiver is implicated, the patient’s dietary compliance increases [27,28,29]. Randomised studies have assessed methods to improve dietary compliance [23]. These studies have mainly focused on educating patients about HF and diet, as well as increasing the frequency of visits with healthcare professionals [30,31]. These interventions improve compliance, with less salt consumed by patients.

Sevilla-Cazes et al. assessed HF self-care from the patient’s perspective [32]. Patients adapted to recommendation rather than compliance, adapting being a process of changing habits: an equilibrium between complying with recommendations and various competing factors. Several factors were found to influence the patient’s adaption, including the lack of clear recommendations. The lack of clear and consistent HF clinical practice guidelines makes it difficult for healthcare professionals to be confident about these recommendations, resulting in diminished clarity for patients.

Currently, healthcare professionals are faced with a dilemma concerning the use of salt-restricted diets for treating HF patients. Firstly, there is clearly a lack of evidence of the benefit of these diets in HF patients. Indeed, the 2021 ESC guidelines highlight the need for evidence concerning the benefit of dietary salt restriction [17]. This has resulted in a lack of consensus concerning the role of salt diets in treating HF patients. Our results also show that recommending restricted-salt diets is associated with increased patient burden, which may contribute to poor quality of life observed. Diet, almost certainly, could improve HF outcomes and quality of life. However, we need more research to determine which diets are most appropriate and to ensure that dietary recommendations do not decrease patients’ quality of life.

Our study has several limitations. First, our primary sources of data were cardiologist- and patient study-specific surveys. The data obtained from these surveys are limited by the expected self-reporting biases, such as recall and social desirability biases, which may have impacted our findings. Second, the information and education provided to participants were not standardised. However, this heterogeneity is representative of the “real-world” situation in contemporary healthcare settings that treat HF patients. Third, a key issue limiting studies that assess restricted-salt diets, including this study, is the ability to accurately estimate patient sodium/salt consumption [33]. Several methods exist, including urinary methods (24 h urine, overnight, and single spot urine collections) and dietary methods (food records, 24 h food recall, and food frequency questionnaires) [33]. We chose to use our validated self-reporting instrument [21] and not a urinary method (24 h urine or spot urine collections). Indeed, urine sodium levels vary in HF patients treated with diuretics [34]. Our instrument proved to be convenient and cost-effective for use in large-scale studies, such as the OFICSel observatory. Fourth, our study population was younger than a classical HF population: the length of the study-specific questionnaire may have unintentionally selected younger patients. Fifth, although we assessed whether burden was related to dietary compliance, we did not collect psychosocial and other data known to influence compliance in HF patients [35]. Finally, the OFICSel observatory was a cross-sectional study that collected and analysed observational data. The study was not designed to assess dietary compliance or the efficacy of diets, or to establish causal relationships between the variables analysed. Further evidence from randomised controlled trials is required.

## 5. Conclusions

HF management often includes salt-restricted diets even if the role of these diets in HF management remains controversial. However, patients do not always understand and comply with these diets. Our study shows that restricting salt in HF patients is associated with increased burden, although a causal relationship still needs to be established. We believe that diets are an important part of HF management. However, we need more research to identify effective diets for HF patients and to provide evidence-based recommendations for both patients and cardiologists.

## Figures and Tables

**Table 1:** Demographic, clinical, and biological characteristics of HF patients enrolled in the OFICSel observatory (*n* = 2822).

Variables	All Patients	
	Patients with Data	Estimate
Demographic data		
Age, years	2729	67.4 (±13.8)
Sex	2818	
Female		840 (29.8)
Male		1978 (70.2)
Living environment	2540	
Urban		1726 (68.0)
Rural		814 (32.0)
Living situation	2779	
Couple		1401 (50.4)
Family		502 (18.1)
Retirement home/community		43 (1.5)
Alone		833 (30.0)
Heart failure history		
Type of HF,	2607	
De novo (<3 months)		427 (16.4)
Chronic		2180 (83.6)
Current HF, stable vs. acute	2577	
Stable		1788 (69.4)
Acute		789 (30.6)
Last acute HF episode (months)	2424	
<3		1068 (44.1)
3–12		535 (22.1)
>12		821 (33.9)
Type of cardiopathy	2639	
Ischemic		1162 (44.0)
Non-ischemic		1262 (47.8)
Valvular		215 (8.1)
Cardiovascular risk factors		
Current smoker	2822	321 (11.4)
Number of cigarettes/day	272	10.0 (5.0; 15.0)
Hypercholesterolemia	2822	1072 (38.0)
Hypertension	2822	1578 (55.9)
Obesity	2822	584 (20.7)
Diabetes	2822	816 (28.9)
Family history of coronary disease	2822	230 (8.15)
Dialysis	2822	17 (0.6)
Sleep apnoea syndrome	2822	231 (8.2)
Patients with chronic obstructive pulmonary disease	2822	199 (7.1)
**Clinical and biological variables**		
NYHA class (physician), *n* (%)	2530	
I		344 (13.6)
II		1215 (48.0)
III		786 (31.1)
IV		185 (7.3)
Self-reported symptoms, *n* (%)	2541	
Asymptomatic		413 (16.3)
Mild exercise symptoms not limiting daily life		880 (34.6)
Symptoms limiting daily life and/or orthopnoea		1248 (49.1)
Weight loss within the last 6 months (kg)	1255	7.0 (±5.7)
BMI (kg/m^2^)	2688	27.1 (±5.9)
Systolic blood pressure (mmHg)	2688	120.2 (±20.7)
Diastolic blood pressure (mmHg)	2710	70.5 (±12.4)
Heart rate (bpm)	2607	73.0 (±16.4)
Sinus rhythm	2822	1742 (61.7)
QRS width (ms)	772	115.6 (±33.5)
LVEF (%)	2680	38.7 (±13.7)
NT-proBNP levels (pg/mL)	1739	1811 (703; 4384)
BNP levels (pg/mL)	828	438 (177; 885)
NT-proBNP and BNP quartiles combined	2448	
Q1		615 (25.1)
Q2		605 (24.7)
Q3		609 (24.9)
Q4		619 (25.3)
Creatinine level (µmol/L)	2677	99.0 (177; 885)
Haemoglobin level (g/L)	2581	12.9 (11.6; 14.2)
Patients with implantable cardioverter defibrillator	2822	725 (25.7)

Results are *n* (%), mean (±standard deviation), or median (interquartile range). BMI, body mass index; BNP, brain natriuretic peptide; HF, heart failure; LVEF, left ventricular ejection fraction; NT-proBNP, *N*-terminal pro–brain-type natriuretic peptide; NYHA, New York Health Association; Q, quartile; SD, standard deviation.

**Table 2:** Therapeutic education, diet regimens prescribed, and adherence to salt diet in patients enrolled in the OFICSel observatory (*n* = 2822).

Variables	All Patients	
	Patients with Data	Estimate
Patients with therapeutic education programme	2822	657 (23.3)
Patient’s frequency of weighing	2750	
Daily		554 (20.1)
Weekly		1014 (36.9)
Monthly		709 (25.8)
Never		473 (17.2)
Diet recommended to the patients *	2822	
Low-salt diet		2618 (92.8)
Water restriction		402 (14.2)
Diabetic diet (carbohydrate-controlled diet)		768 (27.2)
Low-fat diet		1090 (38.6)
Healthcare professional recommending low-salt diet *	2618	
General practitioner		561 (21.4)
Cardiologist		1541 (58.9)
Dietician		626 (23.9)
Nurse		148 (5.7)
Salt diet recommended (SD-R) by healthcare professional (g/day)	2822	
<3		267 (9.5)
3≥ salt <6		915 (32.4)
6		1186 (42.0)
>6		269 (9.5)
Unknown		185 (6.6)
Salt diet understood (SD-U) by patient (g/day)	2822	
<3		516 (18.3)
3≥ salt <6		664 (23.5)
6		719 (25.5)
>6		528 (18.7)
Unknown		395 (14.0)
Estimated salt consumption (g/day)	2822	4.4 (2.8; 6.2)
<3		753 (26.7)
3≥ salt <6		1291 (45.7)
>6		778 (27.6)
Patients compliance with salt diet understood versus estimated salt consumption	2822	
Compliant		933 (33.1)
Overcompliant		969 (34.3)
Undercompliant		525 (18.6)
Unknown		395 (14.0)
BIRD score (maximum score = 48)	2602	8.1 (±8.8)
BIRD score for the 12 items		
On account of my diet, I am not living as I would like, because …		
… every meal is difficult for me	2665	0.7 (±1.0)
… having a meal away from home is complicated	2664	0.9 (±1.2)
… grocery shopping is complicated	2677	0.7 (±1.1)
… it results in additional expenses	2618	0.6 (±1.0)
… I have the impression of being a bother or a burden to those preparing my meals	2671	0.5 (±0.9)
… it makes relationships or activities with friends or family difficult	2640	0.5 (±1.0)
… it makes my leisure activities difficult (favourite pastimes, sports)	2671	0.8 (±1.2)
… it prevents me from travelling, going on vacation	2626	0.8 (±1.3)
… it makes me feel tired, weary, or I lack energy	2648	1.1 (±1.2)
… it is difficult to manage in my workplace/professional activity	2632	0.4 (±0.9)
… it depresses me	2624	0.6 (±1.0)
… it aggravates my health	2611	0.5 (±0.9)
MLHFQ score (maximum score = 105)	2200	35.4 (±24.5)
Physical subscale (maximum score = 40)	2505	16.7 (±11.7)
Emotional subscale (maximum score = 25)	2589	7.7 (±6.6)

Results are *n* (%), mean (±standard deviation), or median (interquartile range). * Multiple responses possible. BIRD, burden scale in restricted diets; IQR, interquartile range; MLHFQ, Minnesota Living with Heart Failure Questionnaire; SD, standard deviation.

**Table 3:** Concordance between salt diet recommended (SD-R), by the healthcare professional, and salt diet understood (SD-U), by the patient.

		Salt Diet Recommended (SD-R) by Healthcare Professional (g/Day), *n* (%)				
		Unknown (*n* = 185)	None/>6 (*n* = 269)	6 (*n* = 1186)	3–6 (*n* = 915)	<3 (*n* = 267)
**Salt diet understood (SD-U) by patient (g/day), *n* (%)**	Unknown (*n* = 395)	55 (29.7)	24 (8.9)	174 (14.7)	112 (12.2)	30 (11.2)
	None/>6 (*n* = 528)	35 (18.9)	163 (60.6)	205 (17.3)	111 (12.1)	14 (5.2)
	6 (*n* = 719)	41 (22.2)	39 (14.5)	445 (37.5)	173 (18.9)	21 (7.9)
	3–6 (*n* = 664)	27 (14.6)	25 (9.3)	226 (19.1)	337 (36.8)	49 (18.4)
	<3 (*n* = 516)	27 (14.6)	18 (6.7)	136 (11.5)	182 (19.9)	153 (57.3)

**Table 4:** Determinants of daily estimated salt consumption (SD-T): results from univariate and multivariate linear regression modelling.

Determinants of Daily Estimated Salt Consumption (SD-T)	Estimated Salt Consumption (g/Day)	Unadjusted Analysis		Multivariate Analysis	
	Raw Mean (SD)	Unadjusted Beta (95% CI)	*p*-Value	Adjusted Beta (95% CI)	*p*-Value
Sex, female vs. male					
No	4.89 (2.52)	0 (ref)	<0.0001	0 (ref)	<0.0001
Yes	4.17 (2.08)	−0.72 (−0.91; −0.52)		−0.65 (−0.86; −0.43)	
Living environment: urban vs. rural					
Rural	4.57 (2.30)	0 (ref)	0.050	0 (ref)	0.035
Urban	4.77 (2.46)	0.20 (0.001; 0.40)		0.23 (0.02; 0.44)	
Living situation					
Couple	4.64 (2.33)	0 (ref)	0.017	0 (ref)	0.001
Family	4.93 (2.51)	0.29 (0.04; 0.54)		−0.01 (−0.28; 0.26)	
Retirement home/community	3.89 (2.22)	−0.74 (−1.48; −0.01)		−1.54 (−2.34; −0.74)	
Alone	4.67 (2.50)	0.04 (−0.17; 0.24)		−0.24 (−0.47; −0.01)	
Chronic vs. de novo HF					
De novo	5.11 (2.54)	0 (ref)	<0.0001	0 (ref)	<0.0001
Chronic	4.60 (2.39)	−0.51 (−0.76; −0.26)		−0.47 (−0.73; −0.21)	
Acute vs. stable HF					
Stable	4.61 (2.32)	0 (ref)	0.009	0 (ref)	0.002
Acute	4.88 (2.61)	0.27 (0.07; 0.47)		0.34 (0.12; 0.57)	
Current smoker					
No	4.56 (2.34)	0 (ref)	<0.0001	0 (ref)	<0.0001
Yes	5.58 (2.79)	1.02 (0.74; 1.30)		0.63 (0.33; 0.94)	
NT-proBNP and BNP quartiles combined					
Q1	4.90 (2.54)	0 (ref)	<0.0001	0 (ref)	<0.0001
Q2	4.79 (2.39)	−0.10 (−0.37; 0.16)		−0.22 (−0.50; 0.06)	
Q3	4.50 (2.39)	−0.40 (−0.67; −0.13)		−0.44 (−0.72; −0.16)	
Q4	4.28 (2.28)	−0.62 (−0.89; −0.35)		−0.67 (−0.96; −0.38)	
Cardiologist					
No	5.15 (2.43)	0 (ref)	<0.0001	0 (ref)	0.022
Yes	4.28 (2.34)	−0.87 (−1.05; −0.69)		−0.27 (−0.50; −0.04)	
Salt diet understood (SD-U) by patient (g/day)					
Unknown	4.79 (2.58)	0 (ref)	<0.0001	0 (ref)	<0.0001
None or >6 g/day	5.94 (2.20)	1.15 (0.85; 1.45)		0.87 (0.51; 1.24)	
6 g/day	5.04 (2.27)	0.25 (−0.03; 0.53)		0.36 (0.03; 0.69)	
3–6 g/day	4.16 (2.16)	−0.63 (−0.92; −0.35)		−0.48 (−0.82; −0.14)	
<3 g/day	3.44 (2.25)	−1.35 (−1.65; −1.05)		−1.16 (−1.51; −0.80)	
Patients’ frequency of weighing					
None	5.39 (2.54)	0 (ref)	<0.0001	0 (ref)	<0.0001
Daily	3.86 (2.16)	−1.53 (−1.82; −1.24)		−1.19 (−1.52; −0.86)	
Weekly	4.46 (2.25)	−0.93 (−1.19; −0.68)		−0.79 (−1.08; −0.50)	
Monthly	5.19 (2.46)	−0.19 (−0.47; 0.08)		−0.35 (−0.66; −0.05)	
Patients with therapeutic education programme					
No	4.82 (2.42)	0 (ref)	<0.0001	0 (ref)	0.004
Yes	4.21 (2.34)	−0.61 (−0.82; −0.40)		−0.35 (−0.59; −0.11)	

BNP, B-type natriuretic peptide; CI, confidence interval; HF, heart failure; NT-proBNP, *N*-terminal pro–brain-type natriuretic peptide; OR, odds ratio; Q, quartile; ref, reference variable; SD, standard deviation.

**Table 5:** Determinants of the burden associated with salt diet understood according to BIRD scores: results from univariate and multivariate linear regression modelling.

BIRD Score	Lowest to Medium Burden	Highest Burden	Unadjusted Analysis		Multivariate Analysis	
	Q1 to Q3	Q4				
N	1983	619				
Factors Assessed	Raw Estimate	Raw Estimate	Unadjusted OR (95% CI)	*p*-Value	Adjusted OR (95% CI)	*p*-Value
Age, years, mean (SD)	67.2 (13.5)	65.8 (14.5)	0.99 (0.99; 1.00)	0.028	0.98 (0.97; 0.99)	<0.0001
Sex, females vs. males, *n* (%)	535 (27.0)	219 (35.4)	1.48 (1.22; 1.79)	<0.0001	1.65 (1.28; 2.13)	<0.0001
Living environment urban vs. rural, *n* (%)	1183 (65.7)	421 (74.5)	1.52 (1.23; 1.89)	<0.0001	1.64 (1.26; 2.12)	<0.0001
Acute vs. stable HF, *n* (%)	466 (25.7)	255 (45.3)	2.40 (1.97; 2.92)	<0.0001	1.52 (1.16; 2.00)	0.003
Diabetes, *n* (%)	521 (26.3)	235 (38.0)	1.72 (1.42; 2.08)	<0.0001	1.72 (1.35; 2.20)	<0.0001
Chronic obstructive pulmonary disease, *n* (%)	113 (5.7)	74 (12.0)	2.25 (1.65; 3.06)	<0.0001	1.57 (1.05; 2.33)	0.026
NYHA class, *n* (%)				<0.0001		<0.0001
I	279 (15.8)	46 (8.1)	1 (ref)		1 (ref)	
II	904 (51.1)	215 (38.1)	1.44 (1.02; 2.04)		1.24 (0.81; 1.91)	
III	478 (27.0)	246 (43.5)	3.12 (2.20; 4.42)		2.52 (1.60; 3.97)	
IV	109 (6.2)	58 (10.3)	3.23 (2.07; 5.04)		2.49 (1.42; 4.39)	
LVEF, %, mean (SD)	39.2 (13.7)	36.0 (13.2)	0.98 (0.98; 0.99)	<0.0001	0.98 (0.97; 0.99)	<0.0001
Haemoglobin level, g/L, mean (SD)	13.0 (1.9)	12.5 (1.9)	0.87 (0.83; 0.91)	<0.0001	0.92 (0.86; 0.98)	0.011
Salt diet recommended (SD-R) by healthcare professional (g/day)				<0.0001		NS
None or >6	205 (10.3)	32 (5.2)	1 (ref)		-	
6	837 (42.2)	262 (42.3)	2.01 (1.35; 2.98)		-	
3–6	645 (32.5)	206 (33.3)	2.05 (1.37; 3.07)		-	
<3	163 (8.2)	89 (14.4)	3.50 (2.22; 5.50)		-	
Unknown	133 (6.7)	30 (4.8)	1.45 (0.84; 2.49)		-	
Salt diet understood (SD-U) by patient (g/day)				<0.0001		0.006
None or >6	402 (20.3)	62 (10.0)	1 (ref)		1 (ref)	
6	500 (25.2)	179 (28.9)	2.32 (1.69; 3.19)		2.14 (1.40; 3.26)	
3–6	499 (25.2)	141 (22.8)	1.83 (1.32; 2.54)		1.88 (1.21; 2.94)	
<3	338 (17.0)	156 (25.2)	2.99 (2.16; 4.15)		2.19 (1.39; 3.46)	
Unknown	244 (12.3)	81 (13.1)	2.15 (1.49; 3.11)		2.12 (1.32; 3.41)	
Estimated salt consumption (g/day)				<0.0001		0.027
>7	333 (16.8)	102 (16.5)	1.53 (1.13; 2.08)		1.38 (0.92; 2.05)	
5–7	514 (25.9)	103 (16.6)	1 (ref)		1 (ref)	
3–5	646 (32.6)	207 (33.4)	1.60 (1.23; 2.08)		1.50 (1.07; 2.09)	
<3	490 (24.7)	207 (33.4)	2.11 (1.61; 2.75)		1.72 (1.20; 2.45)	

BIRD, burden scale in restricted diets; CI, confidence interval; HF, heart failure; LVEF, left ventricular ejection fraction; NYHA, New York Heart Association; OR, odds ratio; Q, quartile; ref, reference variable; SD, standard deviation; NS: not significant. ref: variable used to compare to the other.

## Data Availability

The data are available upon reasonable request.

## Supplementary Materials

The following are available online at https://www.mdpi.com/article/10.3390/nu14020308/s1, Table S1: Patient-reported outcomes according to the level of burden associated with salt diet as assessed by the BIRD score, Table S2: Factors associated with disagreement between salt diet recommended (SD-R) and that understood (SD-U): patient either underestimated the recommendation, for example patient understands 3–6 g whereas <3 g was recommended, or overestimated the recommendation, for example patient understands <3 g whereas 3–6 g was recommended, Table S3: Determinants of daily estimated salt consumption (SD-T): detailed results from univariate linear regression modelling (*n* = 2822), Table S4: Determinants of the burden (SD-B) associated with salt diet according to BIRD score: detailed results from univariate logistic regression modelling analysis.

## References

[1] Ponikowski P., Anker S.D., Alhabib K., Cowie M.R., Force T.L., Hu S., Jaarsma T., Krum H., Rastogi V., Rohde L.E. (2014). Heart failure: Preventing disease and death worldwide. ESC Heart Fail..

[2] Savarese G., Lund L.H. (2017). Global public health burden of heart failure. Card. Fail. Rev..

[3] Colin-Ramirez E., Ezekowitz J. (2016). Salt in the diet in patients with heart failure. Curr. Opin. Cardiol..

[4] Albert C., Estep J.D. (2019). Economic impact of chronic heart failure management in today’s cost-conscious environment. Card. Electrophysiol. Clin..

[5] Dunlay S.M., Shah N.D., Shi Q., Morlan B., VanHouten H., Long K.H., Roger V.L. (2011). Lifetime costs of medical care after heart failure diagnosis. Circ. Cardiovasc. Qual. Outcomes.

[6] Ramirez E.C., Arcand J., Woo E., Brum M., Morgan K., Christopher W., Velázquez L., Sharifzad A., Feeney S., Ezekowitz J.A. (2019). Design and region-specific adaptation of the dietary intervention used in the sodium-hf trial: A multicentre study. CJC Open.

[7] Spineti P.P.D.M. (2019). Evaluating sodium restriction in heart failure. Arq. Bras. Cardiol..

[8] Khan M.S., Jones D.W., Butler J. (2020). Salt, no salt, or less salt for patients with heart failure?. Am. J. Med..

[9] Ezekowitz J.A., O’Meara E., McDonald M.A., Abrams H., Chan M., Ducharme A., Giannetti N., Grzeslo A., Hamilton P.G., Heckman G.A. (2017). 2017 comprehensive update of the canadian cardiovascular society guidelines for the management of heart failure. Can. J. Cardiol..

[10] Parrinello G., Di Pasquale P., Licata G., Torres D., Giammanco M., Fasullo S., Mezzero M., Paterna S. (2009). Long-term effects of dietary sodium intake on cytokines and neurohormonal activation in patients with recently compensated congestive heart failure. J. Card. Fail..

[11] Paterna S., Parrinello G., Cannizzaro S., Fasullo S., Torres D., Sarullo F., Di Pasquale P. (2009). Medium term effects of different dosage of diuretic, sodium, and fluid administration on neurohormonal and clinical outcome in patients with recently compensated heart failure. Am. J. Cardiol..

[12] Paterna S., Gaspare P., Fasullo S., Sarullo F., Di Pasquale P. (2008). Normal-sodium diet compared with low-sodium diet in compensated congestive heart failure: Is sodium an old enemy or a new friend?. Clin. Sci..

[13] Gupta D., Georgiopoulou V.V., Kalogeropoulos A., Dunbar S.B., Reilly C., Sands J.M., Fonarow G., Jessup M., Gheorghiade M., Yancy C. (2012). Dietary sodium intake in heart failure. Circulation.

[14] McMurray J.J., Adamopoulos S., Anker S.D., Auricchio A., Böhm M., Dickstein K., Falk V., Filippatos G., Fonseca C., Gomez-Sanchez M.A. (2012). ESC guidelines for the diagnosis and treatment of acute and chronic heart failure 2012: The task force for the diagnosis and treatment of acute and chronic heart failure 2012 of the european society of cardiology. Developed in collaboration with the heart failure association (hfa) of the esc. Eur. Heart J..

[15] Yancy C.W., Jessup M., Bozkurt B., Butler J., Casey D.E., Drazner M.H., Fonarow G.C., Geraci S.A., Horwich T., Januzzi J.L. (2013). 2013 ACCF/AHA guideline for the management of heart failure: A report of the american college of cardiology foundation/american heart association task force on practice guidelines. J. Am. Coll. Cardiol..

[16] Ponikowski P., Voors A.A., Anker S.D., Bueno H., Cleland J.G.F., Coats A.J.S., Falk V., González-Juanatey J.R., Harjola V.-P., Jankowska E.A. (2016). 2016 ESC guidelines for the diagnosis and treatment of acute and chronic heart failure: The task force for the diagnosis and treatment of acute and chronic heart failure of the European Society of Cardiology (ESC). Developed with the special contribution of the Heart Failure Association (HFA) of the ESC. Eur. Heart J..

[17] McDonagh T.A., Metra M., Adamo M., Gardner R.S., Baumbach A., Böhm M., Burri H., Butler J., Čelutkienė J., Chioncel O. (2021). 2021 ESC Guidelines for the diagnosis and treatment of acute and chronic heart failure. Eur. Heart J..

[18] Atherton J.J., Sindone A., De Pasquale C., Driscoll A., MacDonald P.S., Hopper I., Kistler P., Briffa T., Wong J., Abhayaratna W. (2018). National heart foundation of australia and cardiac society of australia and new zealand: Guidelines for the prevention, detection, and management of heart failure in Australia 2018. Heart Lung Circ..

[19] Audureau E., Guellich A., Guéry E., Canouï-Poitrine F., Benedyga V., Duchossoir H., Taieb C., Damy T. (2018). Development and validation of a new tool to assess burden of dietary sodium restriction in patients with chronic heart failure: The BIRD questionnaire. Nutrients.

[20] De Tejada M.G.-S., Bilbao A., Ansola L., Quirós R., García-Perez L., Navarro G., Escobar A. (2019). Responsiveness and minimal clinically important difference of the Minnesota living with heart failure questionnaire. Heal. Qual. Life Outcomes.

[21] Duchossoir H.B., Audureau E., Taieb C. (2016). Un nouvel outil diététique pour évaluer les apports sodés d’un patient. Inf. Diét..

[22] Naveiro-Rilo J.C., Diez-Juárez D.M., Blanco A.R., Rebollo-Gutiérrez F., Rodríguez-Martínez A., Rodriguez-Garcia M.A. (2010). Validation of the Minnesota living with heart failure questionnaire in primary care. Rev. Esp. Cardiol..

[23] Van Der Wal M.H., Jaarsma T., Van Veldhuisen D.J. (2005). Non-compliance in patients with heart failure; how can we manage it?. Eur. J. Heart Fail..

[24] Marti C.N., Georgiopoulou V.V., Giamouzis G., Cole R.T., Deka A., Tang W.W., Dunbar S.B., Smith A.L., Kalogeropoulos A.P., Butler J. (2012). Patient-reported selective adherence to heart failure self-care recommendations: A prospective cohort study: The atlanta cardiomyopathy consortium. Congest. Heart Fail..

[25] Matsuoka S., Tsuchihashi-Makaya M., Kayane T., Yamada M., Wakabayashi R., Kato N.P., Yazawa M. (2016). Health literacy is independently associated with self-care behavior in patients with heart failure. Patient Educ. Couns..

[26] Lennie T.A., Moser D.K., Chung M.L. (2020). Insight into differences in dietary sodium adherence between men and women with heart failure. J. Cardiovasc. Nurs..

[27] Dunbar S.B., Clark P.C., Quinn C., Gary R.A., Kaslow N.J. (2008). Family influences on heart failure self-care and outcomes. J. Cardiovasc. Nurs..

[28] Dunbar S.B., Clark P.C., Deaton C., Smith A.L., De A.K., O’Brien M.C. (2005). Family education and support interventions in heart failure. Nurs. Res..

[29] Clark A., Spaling M., Harkness K., Spiers J., Strachan P.H., Thompson D., Currie K. (2014). Determinants of effective heart failure self-care: A systematic review of patients’ and caregivers’ perceptions. Heart.

[30] Kuehneman T., Saulsbury D., Splett P., Chapman D.B. (2002). Demonstrating the impact of nutrition intervention in a heart failure program. J. Am. Diet. Assoc..

[31] West A.J., Miller N.H., Parker K.M., Senneca D., Ghandour G., Clark M., Greenwald G., Heller R.S., Fowler M.B., DeBusk R.F. (1997). A Comprehensive management system for heart failure improves clinical outcomes and reduces medical resource utilization. Am. J. Cardiol..

[32] Sevilla-Cazes J., Ahmad F.S., Bowles K.H., Jaskowiak A., Gallagher T., Goldberg L.R., Kangovi S., Alexander M., Riegel B., Barg F. (2018). Heart failure home management challenges and reasons for readmission: A qualitative study to understand the patient’s perspective. J. Gen. Intern. Med..

[33] Colin-Ramirez E., Arcand J., Ezekowitz J.A. (2015). Estimates of dietary sodium consumption in patients with chronic heart failure. J. Card. Fail..

[34] Damman K., Ter Maaten J.M., Coster J.E., Krikken J.A., Van Deursen V.M., Krijnen H.K., Hofman M., Nieuwland W., Van Veldhuisen D.J., Voors A.A. (2020). Clinical importance of urinary sodium excretion in acute heart failure. Eur. J. Heart Fail..

[35] Evangelista L.S., Berg J., Dracup K. (2001). Relationship between psychosocial variables and compliance in patients with heart failure. Heart Lung.

