# Supplementary material for: Prescription, Compliance, and Burden Associated with Salt-Restricted Diets in Heart Failure Patients: Results from the French National OFICSel Observatory
Source: Nutrients. 2022 Jan 12;14(2):308. doi: 10.3390/nu14020308 (PMC8779371; doi:10.3390/nu14020308)
Supplement: Supplementary file 1 [file nutrients-14-00308-s001.zip › nutrients-1491734-supplementary.pdf]

Supplementary tables

Supplemental Table 1. Patient-reported outcomes according to the level of burden associated with salt diet as assessed by the BIRD score

BIRD Score	Lowest to medium burden Q1 to Q3	Highest burden Q4
N	**1983**	**619**
**Factors assessed**	**Mean (±SD)**	**Mean (±SD)**
**Burden scale In Restricted Diets (BIRD) questionnaire**		
BIRD total score (maximum score = 48)	3.9 (±3.9)	21.4 (±6.9)
Detailed items, (each item score from 0-4)		
On account of my diet, I am not living as I would like, because…		
…every meal is difficult for me	0.4 (±0.7)	1.6 (±1.2)
…having a meal away from home is complicated	0.5 (±0.9)	2.0 (±1.4)
…grocery shopping is complicated	0.4 (±0.8)	1.7 (±1.4)
…it results in additional expenses	0.3 (±0.6)	1.6 (±1.2)
…I have the impression of being a bother or a burden to those preparing my meals	0.2 (±0.6)	1.4 (±1.3)
…it makes relationships or activities with friends or family difficult	0.2 (±0.5)	1.6 (±1.2)
…it makes my leisure activities difficult (favourite pastimes, sports)	0.4 (±0.8)	2.1 (±1.4)
…it prevents me from travelling, going on vacation	0.3 (±0.8)	2.2 (±1.4)
…it makes me feel tired, weary or I lack energy	0.6 (±0.9)	2.6 (±1.1)
…it is difficult to manage in my workplace/professional activity	0.1 (±0.5)	1.2 (±1.5)
…it depresses me	0.3 (±0.6)	1.8 (±1.2)
…it aggravates my health	0.2 (±0.6)	1.5 (±1.2)
**MLHFQ score (maximum score = 105)**	29.6 (±22.2)	56.5 (±19.3)
MLHFQ - Physical dimension (maximum score = 40)	14.4 (±11.1)	25.0 (±9.5)
MLHFQ - Emotional dimension (maximum score = 25)	6.2 (±5.8)	13.2 (±5.7)

BIRD, burden scale in restricted diets; IQR, interquartile range; MLHFQ, Minnesota Living with Heart Failure Questionnaire; Q, quartile; SD, standard deviation.

Supplementary Table 2. Factors associated with disagreement between salt diet recommended (SD-R) and that understood (SD-U): patient either underestimated the recommendation, for example patient understands 3-6 g whereas <3 g was recommended, or overestimated the recommendation, for example patient understands <3 g whereas 3-6 g was recommended.

Factors assessed	Agreement		Disagreement						*p*-value OR Disagreement (Patient underestimated) vs. Agreement	*p*-value OR Disagreement (Patient overestimated) vs. Agreement
			Patient underestimated			Patient overestimated				
N	1153		889			626				
	n (%)	Mean	*n* (%)	OR (95% CI)	Mean	*n* (%)	OR (95% CI)	Mean		
Age	1123	66.9	867	1.01 (1.00;1.02)	68.6	600	1.00 (0.99;1.01)	66.5	**0.005**	0.589
BMI (kg/m^2)	1095	26.9	835	1.01 (0.99;1.03)	27.2	598	1.01 (0.99;1.03)	27.2	0.200	0.295
Systolic blood pressure (mmHg)	1102	120.0	856	1.00 (1.00;1.01)	121.4	597	1.00 (0.99;1.00)	118.5	0.137	0.160
Diastolic blood pressure (mmHg)	1114	70.3	860	1.01 (1.00;1.01)	71.3	603	1.00 (0.99;1.00)	69.7	0.087	0.352
Heart rate (bpm)	1078	73.0	825	1.01 (1.00;1.01)	74.5	574	0.99 (0.98;1.00)	70.4	0.055	**0.002**
LVEF (%)	1105	38.3	841	1.01 (1.00;1.01)	39.5	602	1.00 (0.99;1.01)	38.3	**0.047**	0.958
NT-proBNP levels (pg/mL)	753	3828	562	1.00 (1.00;1.00)	4161	350	1.00 (1.00;1.00)	3216	0.376	0.117
BNP levels (pg/mL)	309	687	255	1.00 (1.00;1.00)	912	224	1.00 (1.00;1.00)	689.7	0.088	0.976
Creatinine levels (µmol/L)	1093	108.7	859	1.00 (1.00;1.00)	103.5	598	1.00 (1.00;1.00)	105.4	0.086	0.285
Haemoglobin (g/L)	1056	12.8	837	1.03 (0.99;1.08)	12.9	574	1.06 (1.01;1.12)	13.0	0.179	**0.021**
QRS (ms)	286	114.5	273	1.00 (1.00;1.01)	116.9	173	1.00 (0.99;1.01)	114.0	0.385	0.875
Weight loss, prior 6 months (kg)	494	7.0	401	0.99 (0.97;1.02)	6.8	294	1.01 (0.98;1.03)	7.3	0.669	0.461
Smoker (number of cigarettes/day)	116	10.1	87	1.03 (1.00;1.07)	12.4	51	1.00 (0.95;1.05)	10.0	0.061	0.874
Salt consumption estimated by the patient (g/day)	1153	4.65	889	1.07 (1.04;1.11)	5.1	626	0.90 (0.86;0.94)	4.06	**0.0001**	**<0.0001**
Sex (female)	357 (44.8)		264 (33.2)	0.94 (0.78;1.14)		175 (22.0)	0.87 (0.70;1.08)		0.548	0.200
Hypertension	654 (43.7)		519 (34.7)	1.07 (0.90;1.28)		323 (21.6)	0.81 (0.67;0.99)		0.452	**0.038**
Current smoker	129 (42.7)		100 (33.1)	1.01 (0.76;1.33)		73 (24.2)	1.05 (0.77;1.42)		0.966	0.764
Sleep apnoea syndrome	100 (45.2)		62 (28.1)	0.79 (0.57;1.10)		59 (26.7)	1.10 (0.78;1.54)		0.160	0.596
Diabetes	341 (44.5)		255 (33.2)	0.96 (0.79;1.16)		171 (22.3)	0.89 (0.72;1.11)		0.661	0.315
Family history of coronary disease	95 (43.2)		78 (35.5)	1.07 (0.78;1.46)		47 (21.4)	0.90 (0.63;1.30)		0.667	0.587
Dialysis	5 (33.3)		4 (26.7)	1.04 (0.28;3.88)		6 (40.0)	2.22 (0.68;7.31)		0.956	0.189
Hypercholesterolemia	440 (43.3)		332 (32.6)	0.97 (0.81;1.16)		245 (24.1)	1.04 (0.85;1.27)		0.706	0.686
Obesity	227 (41.1)		181 (32.8)	1.04 (0.84;1.30)		144 (26.1)	1.22 (0.96;1.54)		0.706	0.101

Chronic obstructive pulmonary disease	97 (50.8)	55 (28.8)	0.72 (0.51;1.01)	39 (20.4)	0.72 (0.49;1.06)	0.058	0.099
NYHA (physician)							
I	133 (41.2)	93 (28.8)	1(ref)	97 (30.0)	1(ref)	0.258	**0.0002**
II	487 (41.9)	377 (32.5)	1.11 (0.82;1.49)	297 (25.6)	0.84 (0.62;1.13)	0.502	0.241
III	321 (43.1)	275 (37.0)	1.23 (0.90;1.67)	148 (19.9)	0.63 (0.46;0.88)	0.199	**0.006**
IV	96 (53.0)	59 (32.6)	0.88 (0.58;1.34)	26 (14.4)	0.37 (0.22;0.62)	0.546	**0.0001**
Chronic vs. *de novo* HF	896 (43.4)	687 (33.3)	0.90 (0.71;1.15)	482 (23.3)	1.09 (0.83;1.45)	0.412	0.527
Acute HF	324 (43.4)	278 (37.3)	1.17 (0.96;1.42)	144 (19.3)	0.76 (0.61;0.96)	0.112	**0.020**
Last acute HF episode (decompensation) (months)							
3-12	202 (39.9)	171 (33.8)	1(ref)	133 (26.3)	1(ref)	0.377	**0.009**
<3	466 (45.1)	348 (33.7)	0.88 (0.69;1.13)	219 (21.2)	0.71 (0.54;0.94)	0.319	**0.015**
>12	311 (39.8)	267 (34.2)	1.01 (0.78;1.32)	203 (26.0)	0.99 (0.75;1.31)	0.916	0.952
Type of cardiopathy							
Ischemic	482 (43.5)	350 (31.6)	1(ref)	277 (25.0)	1(ref)	0.130	0.163
Non ischemic	535 (44.2)	413 (34.1)	1.06 (0.88;1.28)	263 (21.7)	0.86 (0.69;1.05)	0.524	0.143
Valvular	73 (36.9)	76 (38.4)	1.43 (1.01;2.03)	49 (24.7)	1.17 (0.79;1.73)	**0.043**	0.436
Sinus rhythm	728 (43.6)	537 (32.2)	0.89 (0.74;1.07)	403 (24.2)	1.06 (0.86;1.29)	0.207	0.605
Implantable cardioverter defibrillator	299 (43.8)	201 (29.5)	0.83 (0.68;1.02)	182 (26.7)	1.17 (0.94;1.45)	0.084	0.154
Salt diet understood (SD-U) by patient (g/day)							
Unknown	55 (14.8)	316 (85.2)	1(ref)	0 (0.00)	1(ref)	**<0.0001**	**<0.0001**
<3	153 (31.3)	0 (0.0)	1.00 (1.00;1.00)	336 (68.7)	1.00 (1.00;1.00)	.	.
3-6	337 (52.9)	49 (7.7)	0.03 (0.02;0.04)	251 (39.4)	0.34 (0.26;0.44)	**<0.0001**	**<0.0001**
6	445 (65.6)	194 (28.6)	0.08 (0.05;0.11)	39 (5.8)	0.04 (0.03;0.06)	**<0.0001**	**<0.0001**
None or >6	163 (33.1)	330 (66.9)	0.35 (0.25;0.50)	0 (0.0)	1.00 (1.00;1.00)	**<0.0001**	.
Restricted salt diet recommended	967 (40.9)	866 (36.6)	29.02 (11.86;70.99)	533 (22.5)	1.14 (0.86;1.53)	**<0.0001**	0.361
Restricted salt diet recommended by:							
General practitioner	271 (50.8)	130 (24.4)	0.56 (0.44;0.70)	132 (24.8)	0.87 (0.69;1.10)	**<0.0001**	0.245
Cardiologist	685 (46.9)	329 (22.5)	0.40 (0.34;0.48)	447 (30.6)	1.71 (1.38;2.10)	**<0.0001**	**<0.0001**
Dietician	255 (42.9)	142 (23.9)	0.67 (0.53;0.84)	198 (33.3)	1.63 (1.31;2.03)	**0.0005**	**<0.0001**
Nurse	78 (55.3)	35 (24.8)	0.56 (0.38;0.85)	28 (19.9)	0.65 (0.41;1.01)	**0.006**	0.053

Low-fat diet	437 (41.5)	346 (32.8)	1.06 (0.89;1.28)	271 (25.7)	1.28 (1.05;1.56)	0.511	**0.017**
Water restriction	149 (38.3)	126 (32.4)	1.13 (0.87;1.46)	114 (29.3)	1.51 (1.15;1.97)	0.353	**0.003**
Diabetic diet (carbohydrate-controlled diet)	334 (45.4)	238 (32.3)	0.91 (0.75;1.11)	164 (22.3)	0.87 (0.69;1.08)	0.370	0.203
Natriuretic peptides levels (NT-proBNP and BNP) combined in quartile (Q)							
Q1	231 (39.2)	192 (32.6)	1(ref)	166 (28.2)	1(ref)	0.532	**0.044**
Q2	260 (44.9)	182 (31.4)	0.84 (0.64;1.10)	137 (23.7)	0.73 (0.55;0.98)	0.211	**0.034**
Q3	260 (44.4)	197 (33.7)	0.91 (0.70;1.19)	128 (21.9)	0.69 (0.51;0.92)	0.496	**0.011**
Q4	247 (42.4)	205 (35.2)	1.00 (0.77;1.30)	130 (22.3)	0.73 (0.55;0.98)	0.991	**0.036**
Patient's frequency of weighing							
Never	202 (46.7)	162 (37.4)	1(ref)	69 (15.9)	1(ref)	**<0.0001**	**0.0007**
Daily	266 (50.1)	132 (24.9)	0.62 (0.46;0.83)	133 (25.0)	1.46 (1.04;2.06)	**0.001**	**0.030**
Weekly	416 (42.9)	285 (29.4)	0.85 (0.66;1.10)	268 (27.7)	1.89 (1.38;2.58)	0.227	**<0.0001**
Monthly	247 (37.0)	275 (41.2)	1.39 (1.06;1.82)	146 (21.9)	1.73 (1.23;2.43)	**0.017**	**0.002**
Patients with therapeutic education program	286 (45.8)	171 (27.4)	0.72 (0.58;0.89)	168 (26.9)	1.11 (0.89;1.39)	**0.003**	0.348
Live environment, urban vs. rural	741 (45.6)	521 (32.1)	0.75 (0.61;0.91)	362 (22.3)	0.71 (0.57;0.88)	**0.004**	**0.002**
Living situation							
Couple	579 (43.7)	405 (30.6)	1(ref)	341 (25.7)	1(ref)	**0.016**	**0.047**
Family	226 (48.0)	145 (30.8)	0.92 (0.72;1.17)	100 (21.2)	0.75 (0.57;0.98)	0.488	**0.038**
Retirement home/community	18 (45.0)	19 (47.5)	1.51 (0.78;2.91)	3 (7.5)	0.28 (0.08;0.97)	0.220	**0.044**
Alone	321 (40.5)	293 (37.0)	1.30 (1.07;1.60)	178 (22.5)	0.94 (0.75;1.18)	**0.010**	0.603

Supplemental Table 3. Determinants of daily estimated salt consumption (SD-T): detailed results from univariate linear regression modelling (*n* = 2822).

		**Estimated salt consumption (g/day)**	**Unadjusted analysis**	
		**Raw mean (SD)**	**Unadjusted Beta (95% CI)**	***p*-value**
**Demographic data**				
Age			-0.01 (-0.02;0.00)	**0.001**
Sex, female vs. male	No	4.89 (2.52)	0(ref)	**<0.0001**
	Yes	4.17 (2.08)	-0.72 (-0.91;-0.52)	
Living environment urban vs. rural	Rural	4.57 (2.30)	0(ref)	**0.050**
	Urban	4.77 (2.46)	0.20 (0.00;0.40)	
Living situation	Couple	4.64 (2.33)	0(ref)	**0.017**
	Family	4.93 (2.51)	0.29 (0.04;0.54)	

	Retirement home/community	3.89 (2.22)	-0.74 (-1.48;-0.01)	
	Alone	4.67 (2.50)	0.04 (-0.17;0.24)	
**Heart failure history**				
Chronic vs. *de novo* HF	De novo	5.11 (2.54)	0(ref)	**<0.0001**
	Chronic	4.60 (2.39)	-0.51 (-0.76;-0.26)	
Acute vs. stable HF	Stable	4.61 (2.32)	0(ref)	**0.009**
	Acute	4.88 (2.61)	0.27 (0.07;0.47)	
Last acute HF episode, 3-12 months (ref.)	Last acute HF episode months), 3-12 months ref.)	4.34 (2.30)	0(ref)	**0.013**
	<3	4.66 (2.56)	0.32 (0.07;0.56)	
	>12	4.71 (2.26)	0.37 (0.11;0.64)	
Cardiopathy type, ischaemic (ref.)	Cardiopathy type, ischaemic ref.)	4.70 (2.40)	0(ref)	0.581
	Non-ischaemic	4.66 (2.47)	-0.04 (-0.23;0.15)	
	Valvular	4.51 (2.13)	-0.19 (-0.54;0.17)	
**Cardiovascular risk factors**				
Current smoker	No	4.56 (2.34)	0(ref)	**<0.0001**
	Yes	5.58 (±2.79)	1.02 (0.74;1.30)	
Number of cigarettes/day			0.06 (0.02;0.10)	
Hypercholesterolemia	No	4.62 (2.37)	0(ref)	0.115
	Yes	4.77 (2.49)	0.15 (-0.04;0.33)	
Hypertension	No	4.67 (2.42)	0(ref)	0.916
	Yes	4.68 (2.42)	0.01 (-0.17;0.19)	
Obesity	No	4.69 (2.41)	0(ref)	0.419
	Yes	4.60 (2.45)	-0.09 (-0.31;0.13)	
Diabetes	No	4.73 (2.46)	0(ref)	0.054
	Yes	4.54 (2.30)	-0.19 (-0.39;0.00)	
Family history of coronary disease	No	4.67 (2.42)	0(ref)	0.624
	Yes	4.75 (2.38)	0.08 (-0.24;0.41)	
Dialysis	No	4.67 (2.42)	0(ref)	0.860
	Yes	4.78 (1.93)	0.10 (-1.05;1.26)	
Sleep apnoea syndrome	No	4.69 (2.42)	0(ref)	0.223
	Yes	4.49 (2.34)	-0.20 (-0.53;0.12)	
Chronic obstructive pulmonary disease	No	4.63 (2.38)	0(ref)	**<0.0001**
	Yes	5.28 (2.78)	0.65 (0.30;1.00)	
**Clinical and biological variables**				
NYHA class	I	4.88 (2.41)	0(ref)	0.084
	II	4.62 (2.40)	-0.27 (-0.55;0.02)	
	III	4.70 (2.43)	-0.18 (-0.48;0.13)	
	IV	4.35 (2.35)	-0.53 (-0.96;-0.10)	
Self-reported symptoms	No symptom	4.57 (2.30)	0(ref)	0.559
	Mild exercise symptoms not limiting daily life	4.72 (2.39)	0.15 (-0.14;0.43)	
	Symptoms limiting daily life and/or orthopnoea	4.71 (2.45)	0.13 (-0.13;0.40)	
NT-proBNP and BNP quartiles combined	1	4.90 (2.54)	0(ref)	**0.000**
	2	4.79 (2.39)	-0.10 (-0.37;0.16)	
	3	4.50 (2.39)	-0.40 (-0.67;-0.13)	
	4	4.28 (2.28)	-0.62 (-0.89;-0.35)	
Sinus rhythm	No	4.56 (2.36)	0(ref)	**0.045**
	Yes	4.75 (2.45)	0.19 (0.00;0.37)	
Implantable cardioverter defibrillator	No	4.79 (2.44)	0(ref)	**<0.0001**
	Yes	4.35 (2.32)	-0.44 (-0.64;-0.24)	
Weight loss within the last 6 months			-0.03 (-0.05;-0.01)	**0.017**
BMI			0.00 (-0.02;0.01)	0.929

Systolic blood pressure			0.01 (0.01;0.02)	**<0.0001**
Diastolic blood pressure			0.02 (0.01;0.03)	**<0.0001**
Heart rate			0.01 (0.01;0.02)	**<0.0001**
LVEF			0.01 (0.00;0.01)	0.085
NT-proBNP levels			0.00 (0.00;0.00)	**0.033**
BNP levels			0.00 (0.00;0.00)	0.595
Creatinine level			0.00 (0.00;0.00)	**0.024**
Haemoglobin level			0.06 (0.01;0.10)	**0.020**
QRS width			0.00 (-0.01;0.00)	0.105
**Diet-related variables**				
Low-salt diet	No	6.16 (2.34)	0(ref)	**<0.0001**
	Yes	4.56 (2.39)	-1.61 (-1.95;-1.27)	
Healthcare professional recommending low-salt diet				
General practitioner	No	4.70 (2.40)	0(ref)	0.309
	Yes	4.58 (2.49)	-0.12 (-0.34;0.11)	
Cardiologist	No	5.15 (2.43)	0(ref)	**<0.0001**
	Yes	4.28 (2.34)	-0.87 (-1.05;-0.69)	
Dietician	No	4.77 (2.45)	0(ref)	**<0.0001**
	Yes	4.35 (2.27)	-0.41 (-0.63;-0.20)	
Nurse	No	4.66 (2.41)	0(ref)	0.363
	Yes	4.85 (2.55)	0.19 (-0.21;0.59)	
Water restriction	No	4.74 (2.45)	0(ref)	**<0.0001**
	Yes	4.27 (2.19)	-0.47 (-0.73;-0.22)	
Diabetic diet (carbohydrate-controlled diet)	No	4.70 (2.45)	0(ref)	0.399
	Yes	4.61 (2.34)	-0.09 (-0.29;0.11)	
Low-fat diet	No	4.71 (2.43)	0(ref)	0.296
	Yes	4.61 (2.39)	-0.10 (-0.28;0.09)	
Salt diet recommended (SD-R) by healthcare professional	Unknown	4.83 (2.39)	0(ref)	**<0.0001**
	None or >6 g/day	5.89 (2.34)	1.06 (0.62;1.50)	
	6 g/day	4.91 (2.35)	0.08 (-0.28;0.45)	
	3-6 g/day	4.27 (2.35)	-0.56 (-0.93;-0.19)	
	<3 g/day	3.67 (2.40)	-1.16 (-1.60;-0.72)	
Salt diet understood (SD-U) by patient	Unknown	4.79 (2.58)	0(ref)	**<0.0001**
	None or >6 g/day	5.94 (2.20)	1.15 (0.85;1.45)	
	6 g/day	5.04 (2.27)	0.25 (-0.03;0.53)	
	3-6 g/day	4.16 (2.16)	-0.63 (-0.92;-0.35)	
	<3 g/day	3.44 (2.25)	-1.35 (-1.65;-1.05)	
Patient compliance with salt diet understood versus estimated salt consumption	Compliant	4.72 (2.43)	0(ref)	**<0.0001**
	Overcompliant	3.57 (1.59)	-1.14 (-1.34;-0.95)	
	Undercompliant	6.54 (2.36)	1.82 (1.58;2.05)	
	Unknown	4.79 (2.58)	0.07 (-0.18;0.33)	
Patient frequency of weighing	None	5.39 (2.54)	0(ref)	**<0.0001**
	Daily	3.86 (2.16)	-1.53 (-1.82;-1.24)	
	Weekly	4.46 (2.25)	-0.93 (-1.19;-0.68)	
	Monthly	5.19 (2.46)	-0.19 (-0.47;0.08)	
Patients with therapeutic education program	No	4.82 (2.42)	0(ref)	**<0.0001**
	Yes	4.21 (2.34)	-0.61 (-0.82;-0.40)	

BMI, body mass index; BNP, B-type natriuretic peptide; CI, confidence interval; HF, heart failure; IQR, interquartile range; LVEF, left ventricular ejection fraction; NT-proBNP, N-terminal pro–brain-type natriuretic peptide; NYHA, New York Heart Association; OR, odds ratio; Q, quartile; ref, reference variable; SD, standard deviation; SD-R, salt diet recommended; SD-U, salt diet understood.

Supplementary Table 4. Determinants of the burden (SD-B) associated with salt diet according to BIRD score: detailed results from univariate logistic regression modelling analysis.

Factors		Lowest to medium burden BIRD score Q1 to Q3	Highest burden BIRD score Q4	Unadjusted analysis	
N		1983	610		
		Raw estimate	Raw estimate	Unadjusted OR (95% CI)	*p*-value
**Demographic data**					
Age, years, median (SD)		67.3 (13.5)	65.30 (14.5)	0.99 (0.98;1.00)	**0.002**
Sex, female vs. male, *n* (%)		541 (27.2)	213 (34.9)	1.43 (1.18;1.74)	**<0.0001**
Living environment urban vs. rural, n (%)		1187 (65.6)	416 (75.1)	1.58 (1.27;1.96)	**<0.0001**
Living situation	Couple	1015 (51.4)	292 (48.0)	1(ref)	0.457
	Family	364 (18.4)	119 (19.6)	1.14 (0.89;1.45)	
	Retirement home/community	26 (1.3)	11 (1.8)	1.47 (0.72;3.01)	
	Alone	571 (28.9)	186 (30.6)	1.13 (0.92;1.40)	
**Heart failure history**					
Chronic vs. *de novo* HF, n (%)		1539 (83.7)	488 (85.8)	1.17 (0.90;1.53)	0.235
Acute vs. stable HF, *n* (%)		468 (25.7)	252 (45.4)	2.41 (1.98;2.93)	**<0.0001**
Last acute HF episode (months), 3-12 months (ref.), *n* (%)		401 (23.7)	106 (19.5)	1(ref)	**<0.0001**
	<3	663 (39.2)	318 (58.6)	1.81 (1.41;2.34)	
	>12	628 (37.1)	119 (2)	0.74 (0.56;0.99)	
Cardiopathy type, ischaemic (ref.), *n* (%)		804 (43.3)	279 (47.9)	1(ref)	0.136
	Non-ischaemic	906 (48.8)	258 (44.3)	0.82 (0.68;1.00)	
	Valvular	148 (8.0)	46 (7.9)	0.90 (0.63;1.28)	
**Cardiovascular risk factors**					
Current smoker, *n* (%)		226 (11.4)	76 (12.3)	1.09 (0.82;1.44)	0.550
Number of cigarettes/day, mean (SD)		10.6 (8.0)	11.1 (6.9)	1.01 (0.97;1.05)	0.650
Hypercholesterolemia, *n* (%)		734 (37.0)	263 (42.5)	1.26 (1.05;1.51)	0.015
Hypertension, *n* (%)		1065 (53.7)	373 (60.3)	1.31 (1.09;1.57)	**0.004**
Obesity, *n* (%)		406 (20.5)	135 (21.8)	1.08 (0.87;1.35)	0.475
Diabetes, *n* (%)		521 (26.3)	235 (38.0)	1.72 (1.42;2.08)	**<0.0001**
Family history of coronary disease, *n* (%)		167 (8.4)	52 (8.4)	1.00 (0.72;1.38)	0.987
Dialysis, *n* (%)		15 (0.8)	2 (0.3)	0.43 (0.10;1.86)	0.257
Sleep apnoea syndrome, *n* (%)		151 (7.6)	69 (11.1)	1.52 (1.13;2.06)	**0.006**
Chronic obstructive pulmonary disease, *n* (%)		113 (5.7)	74 (12.0)	2.25 (1.65;3.06)	**<0.0001**
**Clinical and biological variables**					
NYHA class, *n* (%)	I	279 (15.8)	46 (8.1)	1(ref)	**<0.0001**
	II	904 (51.1)	215 (38.1)	1.44 (1.02;2.04)	
	III	478 (27.0)	246 (43.5)	3.12 (2.20;4.42)	
	IV	109 (6.2)	58 (10.3)	3.23 (2.07;5.04)	
Self-reported symptoms, *n* (%)	No symptom	334 (18.6)	43 (7.4)	1(ref)	**<0.0001**
	Mild exercise symptoms not limiting daily life	680 (37.8)	151 (25.9)	1.72 (1.20;2.48)	
	Symptoms limiting daily life and/or orthopnoea	783 (43.6)	388 (66.7)	3.85 (2.74;5.41)	
NT-proBNP and BNP quartiles combined, *n* (%)	Q1	468 (27.5)	108 (19.3)	1(ref)	**<0.0001**
	Q2	427 (25.1)	136 (24.3)	1.38 (1.04;1.79)	
	Q3	418 (24.5)	150 (26.8)	1.56 (1.17;2.06)	
	Q4	390 (22.9)	165 (29.5)	1.83 (1.39;2.42)	
Sinus rhythm, *n* (%)		1273 (64.2)	362 (58.5)	0.79 (0.65;0.94)	**0.010**
Implantable cardioverter defibrillator, *n* (%)		499 (25.2)	188 (30.4)	1.30 (1.06;1.58)	**0.010**
Weight loss within the last 6 months, kg, medium (IQR)		5.0 (3.0;10.0)	5.0 (3.0;10.0)	1.00 (0.98;1.02)	0.978

 Confidential

BMI, kg/m^2, mean (SD)		27.2 (6.1)	26.8 (5.7)	0.99 (0.97;1.00)	0.127
Systolic blood pressure, mmHg, mean (SD)		121.0 (20.8)	115.9 (19.5)	0.99 (0.98;0.99)	**<0.0001**
Diastolic blood pressure, mmHg, mean (SD)		70.9 (12.3)	69.0 (12.1)	0.99 (0.98;0.99)	**0.001**
Heart rate, bpm, mean (SD)		72.2 (15.7)	74.3 (16.5)	1.01 (1.00;1.01)	**0.005**
LVEF, %, mean (SD)		39.2 (13.7)	36.0 (13.2)	0.98 (0.98;0.99)	**<0.0001**
NT-proBNP levels, pg/mL, median (IQR)		1574 (621;3856)	2250 (958;5255)	1.00 (1.00;1.00)	**0.015**
BNP levels, pg/mL, median (IQR)		424 (158;832)	471 (224;927)	1.00 (1.00;1.00)	0.101
Creatinine level, µmol/L, median (IQR)		98 (76;127)	103 (80;131)	1.00 (1.00;1.00)	**0.050**
Haemoglobin level, g/L, mean (SD)		13.0 (1.9)	12.5 (1.9)	0.87 (0.83;0.91)	**<0.0001**
QRS width, ms, mean (SD)		115.4 (33.9)	116.3 (31.9)	1.00 (1.00;1.00)	0.770
**Diet-related variables**					
Estimated salt consumption, g/day, mean (SD)		4.8 (2.3)	4.4 (2.6)	0.93 (0.90;0.97)	**0.001**
Low-salt diet, *n* (%)		1829 (92.2)	599 (96.8)	2.52 (1.57;4.05)	**<0.0001**
Healthcare professional recommending low-salt diet, *n* (%)					
General practitioner		372 (18.8)	165 (26.7)	1.57 (1.27;1.94)	**<0.0001**
Cardiologist		1066 (53.8)	400 (64.6)	1.57 (1.30;1.89)	**<0.0001**
Dietician		452 (22.8)	148 (23.9)	1.06 (0.86;1.32)	0.565
Nurse		96 (4.8)	47 (7.6)	1.62 (1.13;2.32)	**0.009**
Water restriction, *n* (%)		287 (14.5)	85 (13.7)	0.94 (0.72;1.22)	0.646
Diabetic diet (carbohydrate-controlled diet), *n* (%)		486 (24.5)	227 (36.7)	1.78 (1.47;2.16)	**<0.0001**
Low-fat diet, *n* (%)		752 (37.9)	275 (44.4)	1.31 (1.09;1.57)	**0.004**
Salt diet recommended (SD-R) by healthcare professional, *n* (%)	Unknown	133 (6.7)	30 (4.8)	1(ref)	**<0.0001**
	None or >6 g/day	205 (10.3)	32 (5.2)	0.69 (0.40;1.19)	
	6 g/day	837 (42.2)	262 (42.3)	1.39 (0.91;2.11)	
	3-6 g/day	645 (32.5)	206 (33.3)	1.42 (0.92;2.17)	
	<3 g/day	163 (8.2)	89 (14.4)	2.42 (1.51;3.88)	
Salt diet understood (SD-U) by patient, *n* (%)	Unknown	244 (12.3)	81 (13.1)	1(ref)	**<0.0001**
	None or >6 g/day	402 (20.3)	62 (10.0)	0.46 (0.32;0.67)	
	6 g/day	500 (25.2)	179 (28.9)	1.08 (0.80;1.46)	
	3-6 g/day	499 (25.2)	141 (22.8)	0.85 (0.62;1.16)	
	<3 g/day	338 (17.0)	156 (25.2)	1.39 (1.01;1.90)	
Patients compliance with salt diet understood (SD-U) versus estimated salt consumption, *n* (%)	Compliant	671 (33.8)	200 (32.3)	1(ref)	0.192
	Overcompliant	703 (35.5)	202 (32.6)	0.96 (0.77;1.20)	
	Undercompliant	365 (18.4)	136 (22.0)	1.25 (0.97;1.61)	
	Unknown	244 (12.3)	81 (13.1)	1.11 (0.83;1.50)	
Patient's frequency of weighing, *n* (%)	Never	339 (17.3)	95 (15.8)	1(ref)	**0.001**
	Daily	368 (18.8)	160 (26.5)	1.55 (1.16;2.08)	
	Weekly	737 (37.7)	212 (35.2)	1.03 (0.78;1.35)	
	Monthly	510 (26.1)	136 (22.6)	0.95 (0.71;1.28)	
Patients with therapeutic education program		486 (24.5)	149 (24.1)	0.98 (0.79;1.21)	0.825

BIRD, burden scale in restricted diets; BMI, body mass index; BNP, B-type natriuretic peptide; CI, confidence interval; HF, heart failure; IQR, interquartile range; LVEF, left ventricular ejection fraction; NT-proBNP, N-terminal pro–brain-type natriuretic peptide; NYHA, New York Heart Association; OR, odds ratio; Q, quartile; ref, reference variable; SD, standard deviation; SD-R, salt diet recommended; SD-U, salt diet understood.
